# Free-Standing Iridescent Films of Cellulose Nanocrystal Doped with Eu^3+^ and Tb^3+^ Ions for Photonic Applications

**DOI:** 10.1021/acsomega.5c02252

**Published:** 2025-06-28

**Authors:** Pedro H. L. Sanches, Molíria V. do Santos, Hernane S. Barud, Sidney J. L. Ribeiro, José Maurício A. Caiut

**Affiliations:** † Departamento de Química, Grupo de Nanomateriais e Sistemas Luminescentes, Faculdade de Filosofia, Ciências e Letras de Ribeirão Preto, 124588 Universidade de São Paulo (USP), 14040-901 Ribeirão Preto, SP, Brazil; ‡ Instituto de Química, 153997 Universidade Estadual Paulista (UNESP), 14800-900 Araraquara, São Paulo, Brazil; § Laboratório de Biopolímeros e Biomateriais, Universidade de Araraquara (Uniara), 14801-320 Araraquara, São Paulo, Brazil

## Abstract

The simultaneous integration of iridescence and light emission into a photonic material is an attractive proposal for designing novel optical devices. These properties could be controlled by the action of chiral nematic liquid crystals, and the self-assembly of cellulose nanocrystals is a smart methodology for the development of this new material. Herein, bacterial cellulose (BC) has been used as a cellulose source, mainly due to its biocompatibility, nontoxicity, and high purity. In this context, cellulose nanocrystals (CNCs), obtained by acid hydrolysis methodology, have received significant interest in the production of new optical materials due to their controllable chiral nematic self-organization. The work aims to obtain new iridescent films based on CNCs with adjustable cholesteric pitch, in the presence of lanthanide ions (Ln^3+^), specifically Eu^3+^ and Tb^3+^ ions, as a new platform for optical systems. The results from scanning electron microscopy (SEM) confirmed the chiral nematic structure of the CNC film, and it was corroborated by the Bragg diffraction band observed at specular reflectance analysis. In addition, the following methodology allows control of the cholesteric pitch. The luminescence results from the spectroscopic probe, Eu^3+^ ion, show the lanthanide in a low symmetry environment. The possibility of the shift of the Bragg band may influence Tb^3+^ ion emission, and as a result, the emission color changes. In conclusion, the ability to control the cholesteric pitch of the doped iridescent CNC films is associated with the angle-dependent excitation wavelength and consequently the emission of the material; these new films show an innovative path with potential applications in polarized luminescence and CNC-based optical devices, sensors, tunable filters, and lasers.

## Graphical abstract

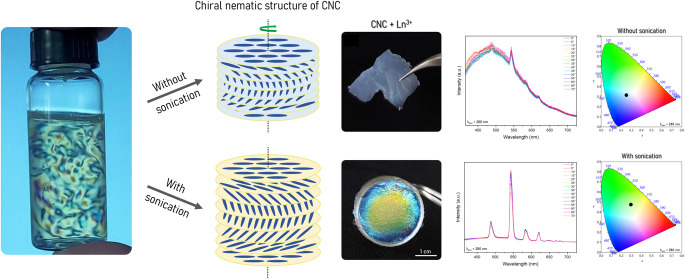

## 1 Introduction

The self-organization of liquid crystals stimulates a broad field of applications and growing interest. Specifically, chiral nematic liquid crystals, which consist of systems organized in a long-range helical orientation, exhibit unique properties such as selective reflection of circularly polarized light, dependent on the angle. This reflection results in the observation of iridescence when the helical pitch is on the order of the wavelength of visible light.^1,2^ For this reason, chiral nematic liquid crystals have been studied for their photonic properties and used in applications such as tunable filters,^3–6^ polarizing mirrors,^7^ reflective displays,^8–10^ and lasers.^11–13^

In recent years, there has been significant interest in the study of cellulose nanocrystals (CNCs), nanoscale fibrils with a large surface area that can exhibit liquid crystal properties.^14–16^ Above a critical CNC concentration in aqueous suspension, the system orders into a chiral nematic phase to minimize existing electrostatic interactions. Interestingly, this configuration can be preserved through slow drying, resulting in iridescent films.^17,18^ In the context of this study, the self-organizing capacity of CNCs has been particularly explored to produce ordered materials as a template capable of controlling the size, structure, and organization of inorganic materials.^19–21^ Stable CNC suspensions can be obtained through the acid hydrolysis of different cellulose sources, including plants,^22–24^ bacteria,^25^ and tunicates.^26^

Specifically, bacterial cellulose (BC) emerges as an interesting cellulose source because it can be obtained via fermentation, requiring only mild alkaline treatment for impurity removal, resulting in high purity and nontoxicity characteristics.^27–29^ By reusing cellulose waste for CNC production and adhering to green processing methods, this study expands the body of research on all-cellulose composites for their technical and economic viability as sustainable alternatives in short-term applications. These materials offer both renewability and biodegradability while achieving performance levels comparable to those of nonrenewable and nonbiodegradable materials.^30,31^

Chiral nematic liquid crystal suspensions, such as CNCs, can be combined with inorganic material precursors to produce self-supported inorganic films with a chiral nematic structure.^32–35^ MacLachlan and co-workers demonstrated that the chiral nematic phase of CNCs can be used to prepare silica photonic crystals and organosilica films.^32,35,36^ By template action from the CNC nematic structure, the authors obtained solid mesoporous structures with a large surface area and helical extension, allowing the creation of tunable photonic structures. As a consequence, these materials were useful to obtain self-supported luminescent iridescent films of CdS quantum dots integrated and encapsulated in mesoporous silica.^37^

In addition, recent studies on the incorporation of rare earth compounds into chiral nematic structures have led to the possibility of circularly polarized excitation and modulated spontaneous emission, as demonstrated by ZrO_2_:Eu^3+^, Y_2_O_3_:Eu^3+^, YVO_4_:Eu^3+^, and AuNCs.^38–41^ For example, upconversion nanoparticles have also been depicted in the preparation of composites combined with chiral CNCs. The chiral properties and upconversion emission were well preserved in the composites, allowing the authors to propose applications in different domains such as security marking of documents, optical memories, and biochemical sensing.^42^

In fact, new photonic active materials could result from the CNC chiral structure and lanthanide ions. But, the lanthanide ions (Ln^3+^) could present an electrostatic interaction with the CNC, and the chiral nematic organization can be perturbed, and iridescent films would not be obtained. To overcome that challenge, in this work, new luminescent iridescent films were prepared by combining a CNC suspension obtained from reusing bacterial cellulose waste with Ln^3+^, such as Eu^3+^ and Tb^3+^ ions. The materials were obtained as self-supported composite films with conserved chiral nematic organization. The structural and optical properties of this new composite were fully characterized, and it was confirmed that the pitch of cellulose nanocrystal organization could be adjusted by a controlled tip sonication treatment on the precursor CNC suspension, creating new possibilities as an innovative platform for optical systems.

## 2 Results and Discussion

### 2.1 Characterization

CNC suspensions were obtained in a concentration of 1.2% (wt %). The birefringence effect, characteristic of the anisotropic liquid crystalline phase of CNCs, can be observed when the suspension is observed under a crossed polarized light (Figure 1d). This phenomenon arises from the variation in the refractive index of CNC along orthogonal directions and, combined with the optical dispersion of the molecules, leads to modulation in the refractive indices.^2^

**Figure 1:**
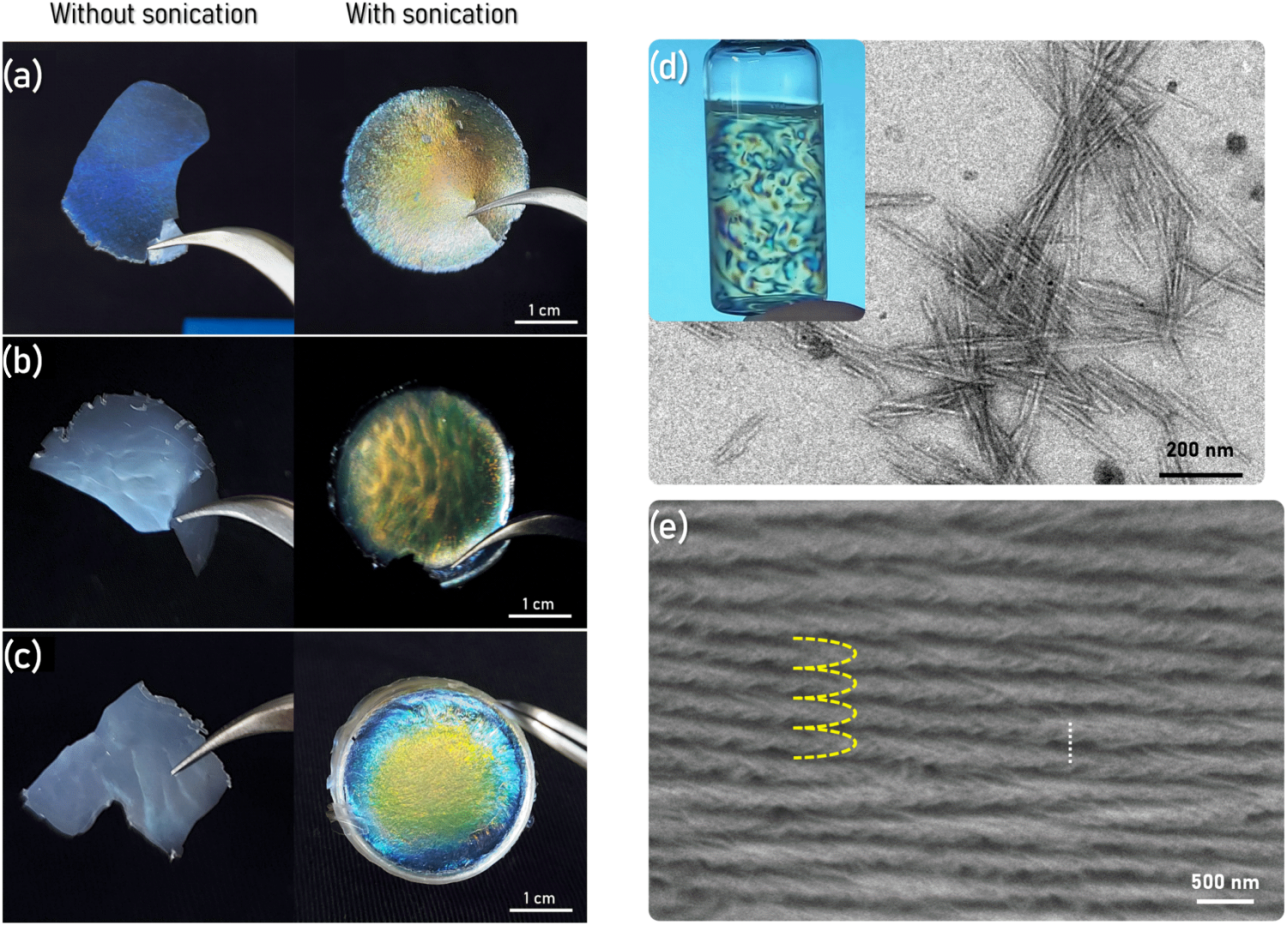
(a) Undoped CNC film, without and with sonication, (b) 1% Eu^3+^-doped CNC film (CNC1%Eu^3+^), without and with sonication, (c) 1% Tb^3+^-doped CNC film (CNC1%Tb^3+^) (wt %), without and with sonication, (d) TEM of CNC suspension (inset photograph from the CNC suspension), and (e) SEM of the fracture of a prepared iridescent film.

TEM images showed the needle-shaped morphology of the nanocrystals (Figure 1d). The dimensions of width and length were comparable to other CNCs obtained from BC extractions according to the literature,^1,28,43,44^ showing an average length of 170.6 ± 34.1 nm and an average width of 9.3 ± 2.4 nm, both within the expected range for H_2_SO_4_ extraction. Additional microscopy images and the distribution of length and width of CNC are shown in Figures S1 and S2 in the Supporting Information. The calculation of the average aspect ratio of the nanocrystals, given by the length divided by the width, is an interesting parameter considering the material’s applicability in reinforcing polymer matrices. Changes in the dimensions of CNCs can significantly alter the interactions between them and modify their ordering within the structures. This characteristic directly influences the formation of the chiral nematic structure.^45,46^ In this case, the obtained value was 18, consistent with the literature for producing materials with better reinforcing properties and satisfactory for cholesteric films.^28,46^

The ζ-potential value for the suspension was −74 mV after the sonication process (further data in Table S1), which aligns with the potential found in the literature for CNC suspensions that resulted in iridescent films, typically ranging between −30 and −70 mV.^43,47,48^ The high ζ-potential values corroborate the CNC stability. Also, the negative surface charge resulted from the presence of sulfate groups on the crystal surface, due to the esterification process of OH groups from cellulose and the tip sonication step, provides enough energy to eject the remaining ionic species as H^+^ close to the CNC surface.^28,49^

The CNC suspension (Figure 1d) resulted in iridescent films of different colors after a slow drying process. The energy provided by the sonication power treatment can modify the pitch of the CNC, thereby changing the wavelength at which incident light will be reflected.^50^ This effect was observed also for the films doped with lanthanide ions such as Eu^3+^ and Tb^3+^ (Figure 1a–c) at concentrations no greater than 1% (wt %). For lanthanide concentration over 1%, the CNC film became opaque due to the electrostatic interaction among Ln^3+^ and CNC particles.

The internal structure of these films was investigated by SEM. Figure 1e shows one cross-sectional image with the pattern of the chiral nematic structure. This pattern is also observed in Bouligand structures, where the rotational organization around an axis promotes different optical and mechanical properties for the materials.^51–53^ The periodicity from this type of structure leads to Bragg reflection, where constructive interference between waves reflected by equivalent planes results in intense reflection of specific wavelengths as a function of the incident light angle. In other words, different wavelengths are reflected and diffracted by different planes according to the angle of incidence of light.^1,51^

The angular dependence of light–film interaction was analyzed by specular reflectance analysis. Films were analyzed by changing the incident light beam angle relative to the normal to the film surface, and an expected behavior of band shifting was observed. For the films *CNC_without sonication* and iridescent *CNC_with sonication* (Figure 2a), a shift of up to 100 nm in the reflectance bands was noted, associated with the angle variation (20° < θ < 60°).

**Figure 2:**
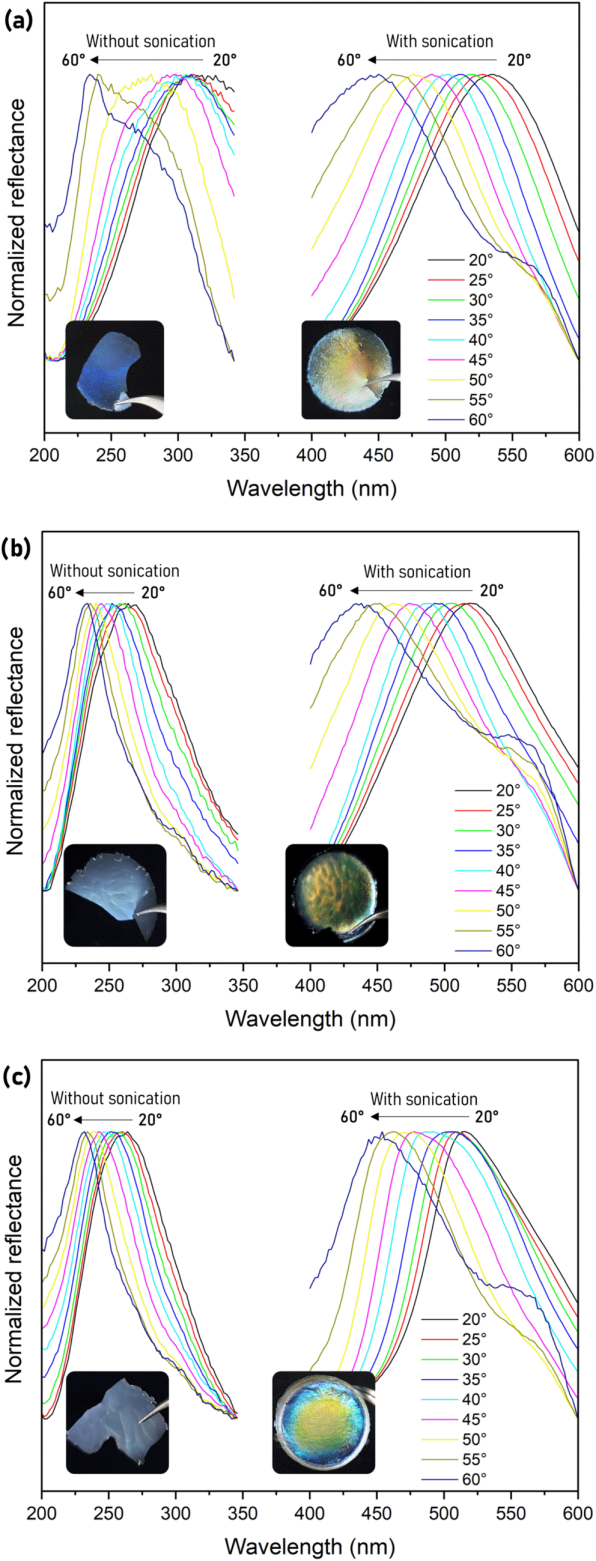
Specular reflectance spectra of films without and with sonication: (a) pure (CNC), (b) doped with 1% Eu^3+^ (CNC1%Eu^3+^), and (c) doped with 1% Tb^3+^ (CNC1%Tb^3+^) (wt %).

For the films *CNC_without sonication*, the reflectance band was observed at the ultraviolet region (Figure 2a). There was a shift in the reflection band toward shorter wavelengths as the incident light angle increased from 320 nm (20°) to 230 nm (60°). In contrast, for the sonicated film, the reflectance band was in the visible region, following the same shifting behavior from 534 nm (20°) to 450 nm (60°).

This effect of shifting the reflectance band is strongly associated with the absorption and reflection of the material, following the behavior described by Vries, where the reflection peak shifts to shorter wavelengths as the incident light angle is increased from the normal to the surface.^52,54^ This relation is described by eq 1 proposed by Vries:

1 $$\lambda_{0}=n_{\mathrm{average}}\cdot P\cdot \sin\theta$$

where λ_0_ is the wavelength of reflection, *n*_average_ is the average refractive index, *P* is the pitch of the chiral nematic structure, and θ is the angle of incidence between the incident ray and the normal to the surface.^54^

The same behavior of the maximum reflection shift toward shorter wavelengths with increasing angle was observed for the iridescent films doped with Eu^3+^ and Tb^3+^ ions (Figure 2b,c). The *CNC1%Eu* ^*3+*^ *_without sonication* film showed a shift from 265 (20°) to 232 nm (60°), while the *CNC1%Eu* ^*3+*^ *_with sonication* film showed a shift from 519 (20°) to 437 nm (60°). As for the *CNC1%Tb* ^*3+*^ *_without sonication* sample, there was a change from 262 nm (20°) to 231 nm (60°), while for the *CNC1%Tb* ^*3+*^ *_with sonication* sample, the shift was from 514 nm (20°) to 452 nm (60°).

Several studies in the literature have already reported on the effect of cations on the pitch of cellulose nanocrystal organization and their iridescence,^2,55–57^ indicating a potential effect of lanthanide ions on the pitch of CNCs. Figure 3 shows that the Bragg diffraction presented a smaller shifting range to lanthanide-doped films compared to undoped materials in the UV range (Figure 3a,b).

**Figure 3:**
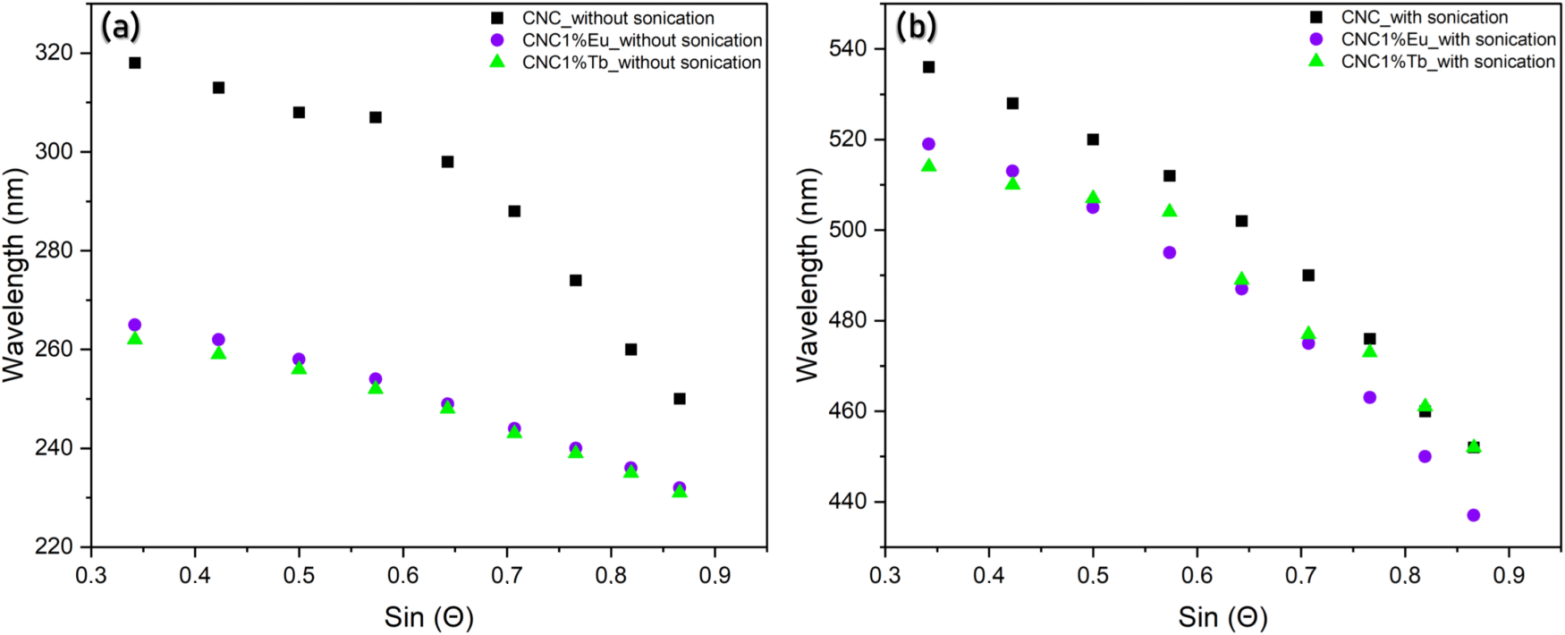
Peak reflection wavelengths of the films prepared (a) without and (b) with the sonication step, as a function of the angles used (sin θ).

The pitch of the nematic phase could be obtained from eq 1,^54^ and the refractive indices of the films were obtained by the M-line prism coupling technique. Table 1 shows the results.

**Table 1:** Index of Refraction (*n*) Values, Calculated Pitch (*P*), and the Variation in the Maximum Reflectance Wavelength (Δλ_max ref_) for Doped and Nondoped Films

**samples**	***n***_**TE**_	***n***_**TM**_	***n***_**average**_	***P* (nm)**	**|Δλ**_**max ref**_ **| (nm)**
*CNC_without sonication*	1.559	1.519	1.539	83.0	68
*CNC1Eu* ^*3+*^ *_ without sonication*	1.563	1.519	1.541	41.7	33
*CNC1Tb* ^*3+*^ *_ without sonication*	1.560	1.520	1.540	38.9	31
*CNC_with sonication*	1.563	1.521	1.542	105.0	111
*CNC1Eu* ^*3+*^ *_with sonication*	1.560	1.519	1.540	100.4	82
*CNC1Tb* ^*3+*^ *_with sonication*	1.558	1.521	1.539	79.1	62

It is important to highlight that the refractive index values were consistent with those found in the literature,^58,59^ close to 1.54. The indices obtained for transverse electric (TE) and transverse magnetic (TM) were different, a typical behavior of materials exhibiting birefringence.^2^

The average refractive indices were consistent with values found in the literature for CNC films from different cellulose sources (1.56,^60^ 1.52,^59^ 1.50,^61^ 1.46 ^62^).

Figure 3a,b clearly shows the effect of the sonication treatment. A red-shift in the reflection wavelength toward longer wavelengths was directly connected to the change in pitch of the cholesteric nematic structure of CNCs. Sonicated films exhibited a higher variation in the maximum reflectance wavelength range compared to films without sonication, consistent with an increase in the calculated cholesteric pitch, and the shift of the reflectance band to longer wavelengths also causes a slight broadening.

In fact, the incident ultrasound can lead to acoustic cavitation and create cycles of compression and rarefaction, resulting in the formation, growth, and collapse of small gas bubbles in the solution. As a consequence, the implosive bubble collapse generates localized hot spots of high temperature and pressure. As a consequence, sonication is an interesting methodology for the synthesis of several categories of materials, e.g., mesoporous TiO_2_ particles.^63^ On the other hand, ultrasound is also used for CNC suspension treatment, and there are several sonication effects on cellulose particles, like physical (size and aspect ratio) and chemical, with the modification of surface charge.^49^ The intense localized forces from sonication may disrupt agglomerates of cellulose nanocrystals (CNCs), resulting in a more homogeneous dispersion. However, the sonication may act on the ions trapped in the bound-water layer of the CNC particles. According to reports in the literature,^28,49^ there is an electrostatic contribution to the increase in pitch by sonication from CNC suspensions, also related to the increase in suspension conductivity. As well-described by Stephanie Beck in her paper,^50^ the bound-water layer surrounds the CNC, and that layer contains residual ions from the hydrolysis process. These act on the electrical double layer and compress it. When the suspension is sonicated, the energy provided to the system can be high enough to eject some ionic species remaining from acid hydrolysis and trapped in the bound-water layer and in the electrical double layer, making them free to diffuse in the suspension. As a result, the size of the electrical double layer increases, and a weaker chiral interaction was observed between particles. The analysis of dynamic light scattering (DLS) was carried out, and the hydrodynamic radius increased after the sonication process. A similar behavior was noted for the ζ-potential, whereas it becomes more negative, as observed in Supporting Information Table S1. Thus, the CNC particles have weaker chiral interactions, leading to a larger pitch, resulting in a shift toward longer wavelengths with the iridescence of the films after drying.^50^

Despite the change in chiral pitch involving external factors from the extraction and drying steps, the doped films exhibited lower pitch values compared to the undoped films (Table 1). This behavior was observed for both films (without or after the sonication treatment), but it was more pronounced in the nonsonicated films. Following the absence of the sonication step in film preparation, the residual ionic species remain on the surface of the nanocrystals; as a consequence, they could interact with the Eu^3+^ or Tb^3+^ ions, resulting in a stronger chiral interaction and affecting the cholesteric pitch. On the other hand, concerning doped and previously sonicated films, the decrease in cholesteric pitch was less pronounced, probably because the residual ions were ejected, and the interaction of the lanthanide into the nanocrystal organization is not stronger enough to influence the chiral interactions and the cholesteric pitch.^40,55,64^

Doped films exhibited a smaller variation in the Δλ_max ref_ compared to nondoped films. This phenomenon can be attributed to the possible effect of increased chiral interaction between the nanocrystals caused by Ln^3+^ ions, which also corroborates the previously discussed decrease in the calculated pitch. This cholesteric pitch behavior was also observed by Frka-Petesic et al.^2^ and Narkevicius et al.^65^ when adding electrolytes (NaCl) to the CNC suspension before drying, resulting in a decrease in cholesteric pitch due to increased ionic strength. Meanwhile, Hirai et al.^66^ observed the aggregation of cholesteric tactoids by adding NaCl, leading to the increased birefringence in suspension and a decrease in the pitch of cellulose nanocrystals.

The chiral nematic structure of CNC stands out as a great potential for photonic applications, and the optical effects depending on the angle of light incidence could be useful for studies of lanthanide spectroscopy and their application. For an initial hypothesis, the excitation or emission of Eu^3+^/Tb^3+^ ions coordinated on the surface of cellulose CNCs could be influenced by the chiral nematic orientation. As shown here, the nematic structure presented a selective light behavior as a function of the incident beam angle, which will affect the lanthanide spectroscopic behavior.

### 2.2 Luminescence

Photoluminescence measurements were conducted to verify any potential changes in the spectral profile based on the incidence angle of the excitation light beam, relative to the normal of the film surface (0° < θ < 70°). Based on previous experiments, the doping concentration was kept at 1 wt.% for all samples because this revealed an optimal luminescence and optical film quality. Higher concentrations led to a decrease in luminescence intensity due to concentration quenching effects.

Excitation spectra for Eu^3+^-containing samples are shown in Figure 4a,d (λ_em_ = 615 nm, ^5^D_0_ → ^7^F_2_). The characteristic excitation ff bands of the europium ion^67,68^ were attributed to the transitions: ^7^F_0_ → ^5^H_3_ (318 nm), ^7^F_0_ → ^5^D_4_ (362 nm), ^7^F_0_ → ^5^L_6_ (394 nm), and ^7^F_0_ → ^5^D_2_ (464 nm). Cellulose is a carbohydrate polymer free of π units but rich in O atoms, so a nonconventional fluorescent property is enhanced in concentrated solutions and solids. That emission could be rationalized by the CTE mechanism (clustering-triggered emission), namely, clustering of nonconventional chromophores as ether, hydroxyl, and carbonyl units, and subsequently electron cloud overlap to form an extended conjugation to rationalize the unique emission behaviors.^69–71^ The cellulose excitation profile was characterized by a broad band from 350 to 500 nm; in addition, a broad band related to the cellulose matrix absorption was observed in the region of 250–300 nm.^72^ These bands were overlapping some Eu^3+^ transitions. Independent of the incident beam angle and the sonication process of the films, the excitation spectral profile was retained; however, the relative intensity changed as a function of the angle from the excitation beam. That effect was more accentuated for the sample *CNC1Eu* ^*3+*^ *_without sonication*, since the Bragg diffraction band for the sample without sonication (Figure 2b) overlaps the excitation spectra (Figure 4a) and possibly the incidence angle of the excitation light beam results in different excitation performance for the cellulose fluorescence.

**Figure 4:**
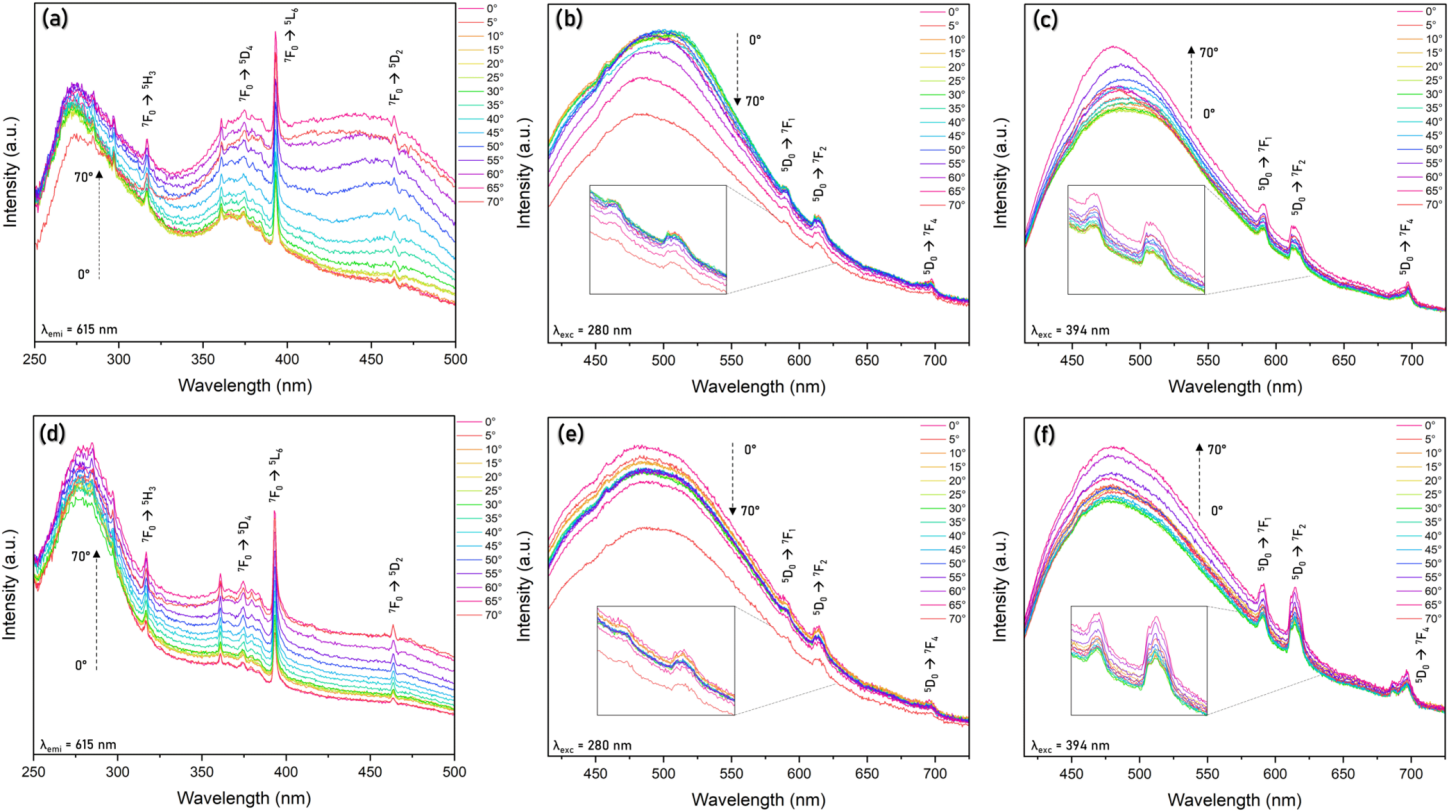
(a) Excitation spectra for the CNC1Eu^3+^_without sonication film (λ_em_ = 615 nm), (b, c) emission spectra for the CNC1Eu^3+^_without sonication film (λ_ex_ = 280 and 394 nm, respectively), (d) excitation spectra for the CNC1Eu^3+^_with sonication film (λ_em_ = 615 nm), and (e, f) emission spectra for the CNC1Eu^3+^_with sonication film (λ_ex_ = 280 and 394 nm, respectively).

Figure 4 also shows emission spectra (4b,c,e,f). The observed Eu^3+^ transitions were ^5^D_0_ → ^7^F_1_ (591 nm), ^5^D_0_ → ^7^F_2_ (615 nm), and ^5^D_0_ → ^7^F_4_ (697 nm) for both sonicated and nonsonicated films (Figure 4b,c,e,f). A broadband emission from the cellulose matrix resulted from the formation of multiple emission centers by clustering states through the CTE mechanism.^69,71^ An analysis of the intensity ratio between the bands ^5^D_0_ → ^7^F_2_/^5^D_0_ → ^7^F_1_ of the samples showed that the intensity of the ^5^D_0_ → ^7^F_1_ transition was closer or even more intense than the ^5^D_0_ → ^7^F_2_ transition, corroborating the Eu^3+^ ion coordinated into a more symmetric environment. Except for the sonicated films, excited at 394 nm (Figure 4f), the emission spectra showed a slightly higher intensity ratio (^5^D_0_ → ^7^F_2_/^5^D_0_ → ^7^F_1_), indicating a decrease in the coordination symmetry.

Concerning the sample *CNC1Eu* ^*3+*^ *_without sonication*, the Bragg reflectance spectra (Figure 2b) are observed to overlap with the emission excitation band. That could result in an excitation filter behavior. The shift in the Bragg peak with the angle (265 nm at 0° and 230 nm at 60°) would lead to a decrease in the filter effect. The emission intensity could be changed by the excitation incident angle; however, that effect was not observed due to the low emission intensity from Eu^3+^ ions, but a slight effect was noted on the cellulose emission.

Lifetime measurements were carried out for two different excitation wavelengths, 394 nm (transition ^7^F_0_ → ^5^L_6_ of Eu^3+^) and 280 nm, without varying the angle (fixed at 45°). The lifetime values (I_0_/e) were 0.18 ms for the *CNC1Eu* ^*3+*^ *_without sonication* film and 0.19 ms for the *CNC1Eu* ^*3+*^ *_with sonication* film (same value for both excitation wavelengths), both consistent with literature data for biologically derived materials with a high concentration of OH groups, which promote the de-excitation of the ^5^D_0_ emitting levels via vibrational mechanisms.^73^ Similar lifetime data were observed for hydrated environment system, as to the Eu^3+^ ion in an aqueous EuCl_3_ solution, presenting a lifetime of 0.14 ms,^74^ or even Eu^3+^ ions coordinated in the lamellar boehmite structure, with a lifetime of 0.16 ms.^74^ Even if the calculated cholesteric pitch showed different values for samples with or without lanthanides (Table 1), the low-intensity emission and lifetime values indicate a Eu^3+^ ion with a hydrated coordination sphere and low interaction with the cellulose structure.

Concerning the Tb^3+^ ions, the excitation spectra from films with or without sonication treatment showed the broad band attributed to the matrix absorption around 250–350 nm with a maximum at 280 nm, an additional contribution of transition 4f5d from Tb^3+^ ions could be expected at higher energy, around 217 nm to aquo ion [Tb­(OH)_2_]_8_,^75^ but no intra f-f excitation bands were noted for the sonicated film (Figure 5c). In addition, the Bragg diffraction band of the sample without sonication (Figure 2c) overlaps the excitation spectra (Figure 5a), and the excitation filter behavior could be corroborated by the low intensity observed in the emission spectra (Figure 5b). Concerning the sonicated film, the Bragg diffraction band was observed in the visible range, so the filter effect did not act on the excitation range. In fact, the Tb^3+^ emission intensity was higher than that of the matrix intrinsic luminescence (Figure 5d). From emission spectra, the characteristic transitions of the Tb^3+^ ion^76^ from ^5^D_4_ → ^7^F_6_ (491 nm), ^5^D_4_ → ^7^F_5_ (547 nm), ^5^D_4_ → ^7^F_4_ (586,5 nm), and ^5^D_4_ → ^7^F_3_ (624 nm) were observed for both films, with a significant change in intensity between sonicated to not sonicated films owing to the iridescence effect and the variation of angles.

**Figure 5:**
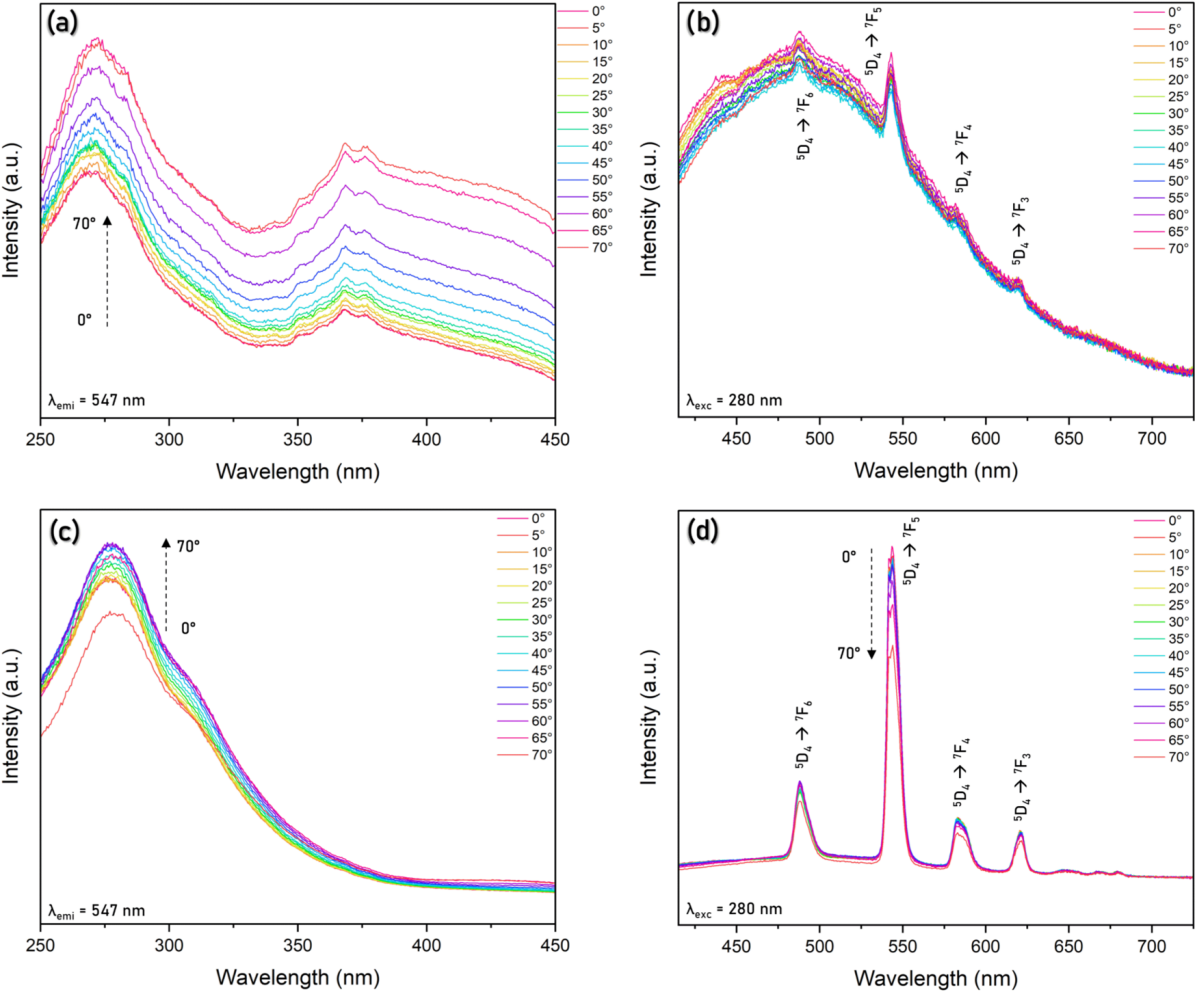
(a) Excitation and (b) emission spectra for the CNC1Tb^3+^_without sonication films. (c) Excitation and (d) emission spectra for the CNC1Tb^3+^_with sonication at different angles.

The sonication process had important effects on the excited-state (^5^D_4_) lifetime. A value of 1.34 ms was observed for excitation at 280 nm for the *CNC1Tb* ^*3+*^ *_with sonication* film. This value is higher than the literature values obtained for materials of biological origin with a high concentration of the OH groups.^73,74^ The *CNC1Tb* ^*3+*^ *_without sonication* film showed a lifetime equal to 0.42 ms.

The inner-filter effect caused by overlapping the Bragg diffraction band with the excitation spectra was more evident in chromaticity diagrams (Figure 6) obtained from the emission spectra. For the film prepared without sonication, the low-efficiency process of the Tb^3+^ ion excitation shifted the emission color to blue and consequentially led to the broadband emission around 400–470 nm, corresponding to the emission of the cellulose matrix (Figure 6a,b). The film with sonication treatment presents the Bragg diffraction band in the visible range, so the filter effect did not act on the excitation range. Then, the emission color was shifted to green and consequentially to a better emission from the Tb^3+^ ion and a low emission from the cellulose matrix. As the filter effect on the excitation was not so evident from the Eu^3+^-doped film, due to the low efficiency of the energy transfer from cellulose to the lanthanide ion, the emission color was not affected (Figure 6c,d).

**Figure 6:**
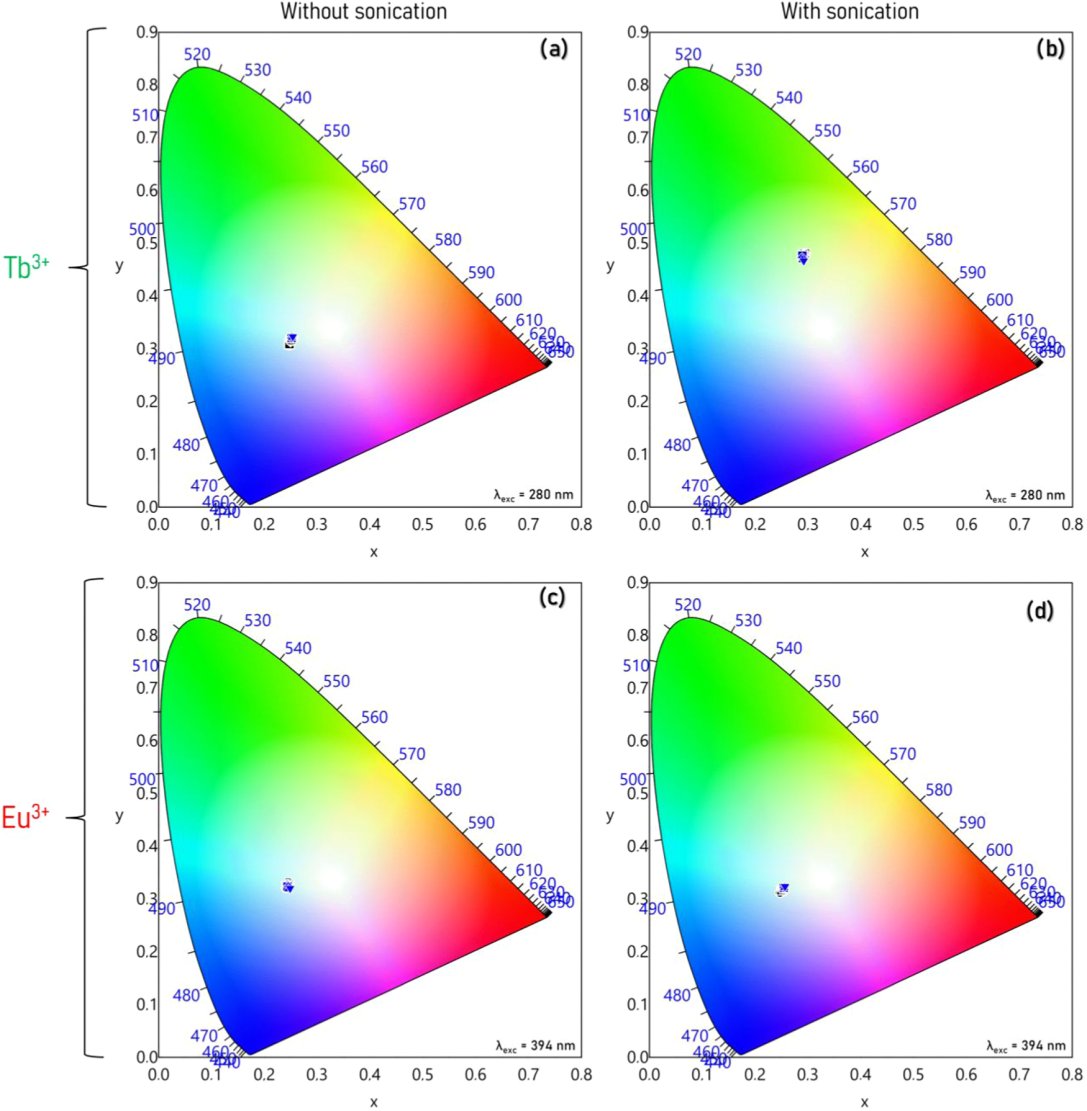
Chromatic diagram as a function of the angle for (a) CNC1Tb^3+^_without sonication film and (b) CNC1Tb^3+^_with sonication film, with emission under 280 nm excitation, and (c) for CNC1Eu^3+^_without sonication film and (d) CNC1Eu^3+^_with sonication film, with emission under 394 nm excitation.

## 3 Conclusions

In summary, we have successfully prepared self-standing films of CNCs derived from bacterial cellulose with the control of the pitch of the chiral nematic organization. The liquid crystal structure was preserved in the presence of lanthanide ions.

Tip sonication treatment was applied to control the pitch of the liquid crystal phase responsible for the Bragg diffraction band observed by the specular reflectance from UV to visible spectra. That property could be useful to control the excitation of lanthanide ions inserted into films, resulting in distinct emission behaviors. The cholesteric structure was also corroborated by scanning electron microscopy; based on the Vries’ equation and the reflectance spectra, the pitch of the organized structure from nanocrystals was calculated. These values confirmed the influence of Eu^3+^ and Tb^3+^ ions on the cholesteric pitch. The self-standing CNC films doped with both Eu^3+^ and Tb^3+^ ions showed a dependence of the emission intensity according to the angle of incidence of the excitation light beam. The effect was clearly observed when the lanthanide excitation wavelength overlapped the reflection band of the cholesteric structure.

The europium luminescence confirmed a low symmetry coordination of the lanthanide into the cholesteric structure, and the lifetime agreed with a hydrophilic environment. Concerning the Tb^3+^-doped film, the influence of angle-dependent light reflection on excitation or emission properties of the utilized ions was also studied. The shift in reflectance band clearly had an inner-filter effect on the excitation spectrum; as a consequence, the color emission changed from blue to green for the Tb^3+^-doped films without or after the sonication treatment on CNC suspension. These results have shown the capability of obtaining a selectively excited luminescent material based on CNC derived from BC-doped lanthanides. In addition, the control of the cholesteric pitch on lanthanides-doped CNC films associated with the emission dependency according to the angle of light incidence, and the selection of excitation wavelength expands the field of applications for this type of material, such as potential sensor applications through signal amplification or attenuation, or even possible photon confinement, important for random lasers studies, e.g., circularly polarized emitters based on the controlled chiral nematic organization and also the adjustable cholesteric pitch.

## 4 Materials and Methods

### 4.1 Materials

High-purity deionized water (18.2 MΩ/cm) was obtained from a Millipore Milli-Q water purification system. The extraction methodology of CNCs was carried out from BC membranes, provided by the HB BIOTEC LTDA,^30,77^ produced by the well-known method of cultivating *Gluconacetobacter xylinum* bacteria in a static culture medium.^78,79^ Sulfuric acid (H_2_SO_4_, Sigma-Aldrich) and sodium hydroxide (NaOH, Sigma-Aldrich) were used. The cellulose membrane for dialysis tubes was from Sigma-Aldrich. For doping the suspensions and films, an aqueous solution of europium chloride (EuCl_3_·6H_2_O) with a concentration of 0.1 mol/L was used. This solution was obtained by digesting europium­(III) oxide (Eu_2_O_3_, Sigma-Aldrich) with hydrochloric acid (HCl, Sigma-Aldrich) and an aqueous solution of terbium chloride (TbCl_3_·6H_2_O, Sigma-Aldrich) in a concentration of 0.1 mol/L.

### 4.2 CNC Extraction

The dried BC membranes were fragmented into pieces of 0.5 cm^2^ and hydrolyzed in a solution of 64% sulfuric acid (H_2_SO_4_) (wt %) (3.47 M) under stirring at 45 °C for 30 min. The hydrolysis was stopped by diluting the mixture to 5 times its volume by adding water at ∼10 °C. The suspension was centrifuged 3 times at 3600 rpm for 15 min and then dialyzed in a cellulose dialysis membrane against ultrapure water until the external water pH reached 5.0. Finally, the suspension was tip sonicated at a ratio of 50 s/1 g of CNC (60% of 300 W power).^28^

### 4.3 Iridescent Film Production

Two different samples could be obtained: (1) films without the previous sonication treatment on CNC suspension and (2) films after the sonication step of the CNC suspension in the ratio of 50 s of sonication per gram of CNC. Films 1 and 2 were named as *CNC_without sonication* and *CNC_with sonication* films, respectively. The total mass to be used was defined as 125 mg of CNC in 10 mL of suspension, which was added to a 3 cm diameter polystyrene Petri dish for slow drying at room temperature.

The Ln^3+^-doped iridescent films were produced by adding 34 μL of the aqueous EuCl_3_ solution (0.1 mol/L) to the CNC suspension to achieve a doping level of 1% Eu^3+^ (wt %) under constant stirring for 30 min. The same procedure was carried out for doping with the Tb^3+^ ion using 33 μL of an aqueous TbCl_3_ solution (0.1 mol/L), followed by drying at room temperature.

### 4.4 Characterization

Powder X-ray diffraction (XRD) analyses were performed using a Bruker AXS D2 phaser diffractometer, between 10–60° and 10–30° (2θ), with a step size and integration time of 0.02°/0.2 s and 0.02°/0.5 s, respectively. UV–vis spectroscopy analyses of the films were conducted using a Thermo Scientific Evolution 60S UV–vis spectrophotometer. Photoluminescence analyses were carried out with a Horiba Scientific FluoroLog 3 (FL3-122) spectrofluorometer, equipped with a double excitation and emission monochromator and a Hamamatsu R928 photomultiplier. A 450 W continuous xenon lamp was used to collect the excitation and emission spectra, and a pulsed lamp was used for lifetime measurements. Specular reflectance analyses were performed using an Agilent Cary 5000 Varian UV/vis/NIR absorption spectrophotometer, varying the angle of incidence of the light beam relative to the normal of the film surface between 20 and 60°. ζ potential analyses were conducted using a ZetaSizer Nano ZS instrument (Malvern). Transmission electron microscopy (TEM) images were acquired by using a JEOL JEM-II microscope. All CNC suspensions were diluted to a concentration of 0.005% (wt %), stained with uranyl acetate, and air-dried on the analysis grids. The length and diameter of the nanocrystals were determined using the free software ImageJ and an average count of 40 particles. Scanning electron microscopy (SEM) images were obtained by using a Shimadzu SS-550 microscope. The imaging utilized the secondary electron (SE) contrast method under a high vacuum at 15 kV, conducted on the cross section of the fractured film in nitrogen, using carbon tape and aluminum support. The refractive indices of the films were obtained by prism coupling the M-line using a wavelength of 632.8 nm, for both transverse electric (TE) and transverse magnetic (TM) polarizations.

## Supplementary Material



## Notes

Transmission electron microscopy images of CNC suspensions; ζ-potential values, dimensions of the hydrodynamic radius obtained by DLS and average length and width calculated from TEM images of the different CNC suspensions; distribution of length of the CNC_with sonication suspension measured from TEM images; and images of undoped CNC films under different sonication treatment and their respective optical microscopies images (PDF)
