# Supplementary material for: Free-Standing Iridescent Films of Cellulose Nanocrystal Doped with Eu3+ and Tb3+ Ions for Photonic Applications
Source: ACS Omega. 2025 Jun 28;10(27):29295–305. doi: 10.1021/acsomega.5c02252 (PMC12268446; doi:10.1021/acsomega.5c02252)
Supplement: Supplementary file 1 [file ao5c02252_si_001.pdf]

Free-standing iridescent films of cellulose nanocrystal doped with Eu^{3+} and Tb^{3+} ions for photonic applications

Pedro H. L. Sanches [1], Molíria V. do Santos [1,2], Hernane S. Barud [3], Sidney J. L. Ribeiro [2], José Maurício A. Caiut [1*]

[1] Departamento de Química, Grupo de Nanomateriais e Sistemas Luminescentes, Faculdade de Filosofia, Ciências e Letras de Ribeirão Preto, Universidade de São Paulo (USP), 14040-901, Ribeirão Preto - SP, Brazil.
[2] Instituto de Química, Universidade Estadual Paulista (UNESP), 14800-900, Araraquara, São Paulo, Brazil.
[3] Laboratório de Biopolímeros e Biomateriais, Universidade de Araraquara (Uniara), 14801-320, Araraquara, São Paulo, Brazil.

Supplementary Information

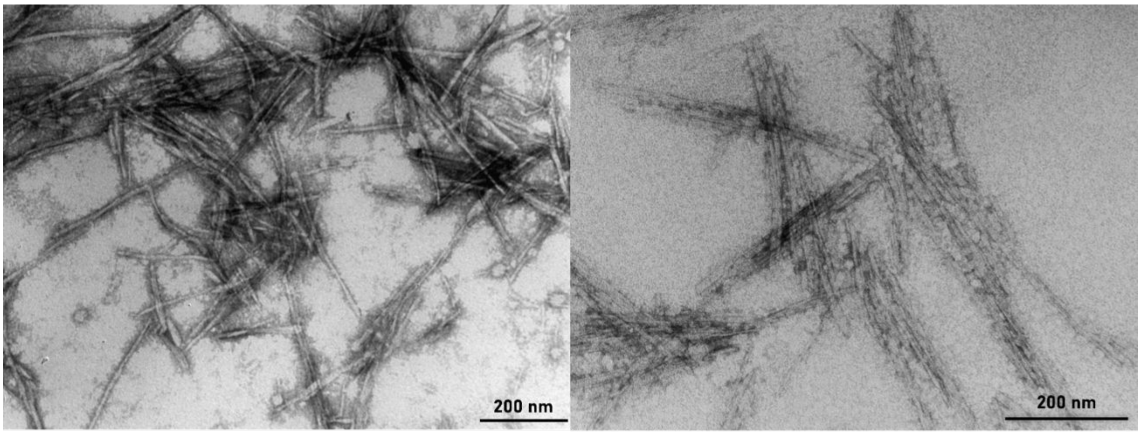

Figure S1 - TEM images of CNC suspensions.

Table S1 - Zeta potential values, dimensions of the hydrodynamic radius obtained by DLS and average length and width calculated from TEM images of the different CNC suspensions.

Samples	Zeta potential (mV)	DLS (nm)	MET average length (nm)	MET average width (nm)
CNC_without sonication	-51,4 ± 1,48	59,3 ± 12,7	-	-
CNC_with sonication	-73,70 ± 1,28	105,8 ± 0,96	170,6 ± 34,7	9,3 ± 2,4

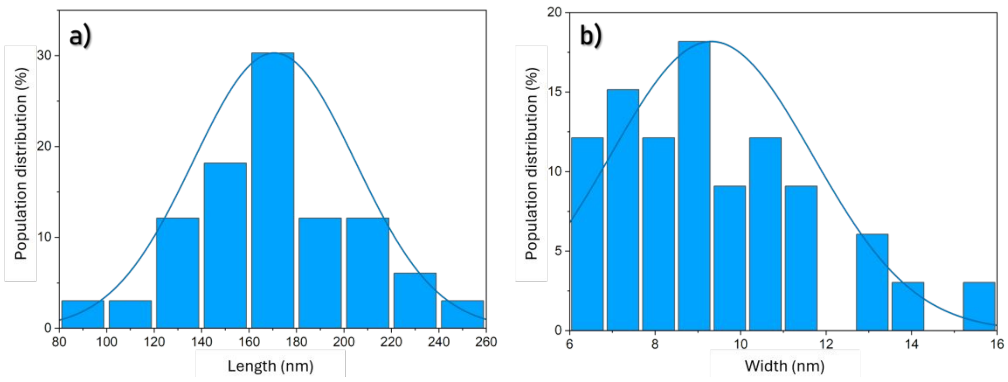

Figure S2 - Distribution of a) length and b) width of *CNC_with sonication* suspension, measured from TEM images with a 40-particle inspection.

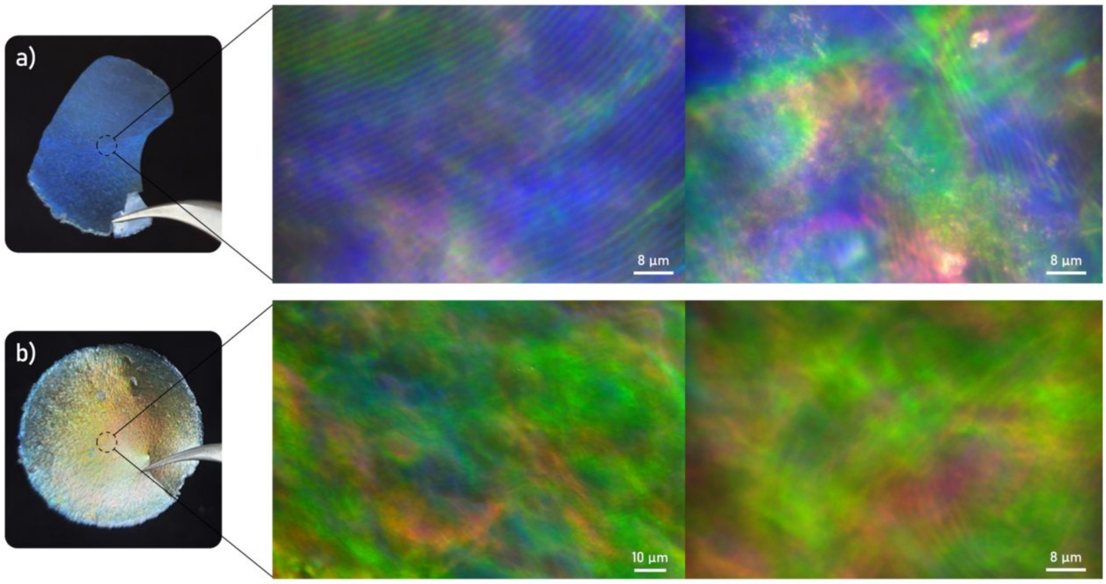

Figure S3 - Undoped CNC films, a) without and b) with sonication, and their respective optical microscopies images on the right side.
